# Meeting Reports

**DOI:** 10.1155/S1463924683000140

**Published:** 1983

**Authors:** K. J. Leiper, Clive J. Jackson

**Affiliations:** 1 Glaxo Operations (UK) Ltd Barnard Castle County Durham DL12 8DT UK; 2 Occupational Hygiene Laboratory 403 Edgware Road London NW2 6LN UK


Journal of Automatic Chemistry, Volume 5, Number 1 (January–March 1983), pages 45–46

### Meeting Reports

### Swansea Summer School of Automated Chemical Analysis—1982

The number of varying interests represented at a course on the topic of laboratory automation is a constant source of amazement and the 1982 Swansea Summer School was no exception. The one factor which welds such a diverse interest group together is the recognition that this is a subject where hypothesis, though of interest, is no substitute for hard practical experience.

This forum provided an interchange of ideas between extremes, academics and people at the sharp end, beginners and the experienced, where delegates could also share their ideas for the future. For some it was a confirmation that their ideas were substantially in the mainstream of opinion, others will now be reassessing their projects, but all will have learned something new.

The only drawback of such a course is the organization of the practical sessions with delegates of differing levels of appreciation and interest in the topics provided. This was overcome by a series of practical sessions and tutorials to supplement the lectures and, though not perfect, my information is that the organizers are aware of the drawbacks and will be incorporating further refinements for next year.

**K. J. Leiper**
*Glaxo Operations (UK) Ltd, Barnard Castle, County Durham DL12 8DT, UK*

### Automatic Sampling, Sample Preparation and Presentation

A joint symposium on the application of robots and more conventional automation to the problems of sampling, sample preparation, and presentation to an appropriate analytical device, was organized by the Automatic Methods Group and the North East. Region of the Analytical Division of the Royal Society of Chemistry and held in the superb surroundings of Wentworth College, York University from 30 September to 1 October 1982. Four lecture sessions were held: on 'Robotics' and on 'Organic, Pharmaceutical and Inorganic applications'.

In the first session on robotics, Dr J. H. W. Cramp discussed the feasibility of using low-cost robots in an analytical laboratory, based on his experiences at ICI's Mond Division. P. Warren (ICI, Petrochemical & Plastics Division) then considered an application of a microprocessor-controlled cartesian co-ordinate robot for sampling and sample presentation of granular plastics to an XRF spectrometer and contrasted this mode of operation with that of a more conventional purpose-built automated system for the same application. Dr T. B. Pierce (AERE, Harwell), giving the keynote lecture, discussed the operation of a variety of laboratory robots and compared the performance and economics of a number of anthropomorphic (jointed arm) robots. Mr J. Parkin (Peerless Systems) then considered applications concerning the weighing-out and preparation of flux beads for XRF analysis. Finally, Mr P. Evins (IBM [UK] Ltd) discussed the impact that robots were making within his company.

The afternoon session on organic applications was opened with a keynote lecture by Dr R. H. Brown (Health and Safety Executive's [HSE] Occupational Hygiene Laboratory) who spoke on the automated processing of organic airborne pollutant samples collected on solid-adsorber tubes. Mr M. House (Perkin-Elmer Ltd) then discussed the development of an automated headspace sample presentation system for gas chromatography and considered its application to the analysis of materials as diverse as beverage aromas and flavours, lubricating oils, polymers and resins, perfumes in detergents and soaps, and a range of microbiological materials. Mr S. Coverly (Technicon Instruments Co. Ltd) considered a range of automated sample-preparation procedures, such as continuous filtration, ion exchange, and evaporation and subsequent re-dissolution. These are used in a fully-automated sample preparation/liquid chromatography ('FAST-LC') system developed by Technicon and a number of examples, ranging from the analysis of water-soluble vitamins, drugs in serum and pesticide residues in food, were given. Finally, Mr C. J. Warwick (HSE's Occupational Hygiene Laboratory) described work that he had been undertaking on the development of a decision-making capability associated with an automated HPLC system to enable optimum analytical procedures to be developed automatically.

The next morning saw two sessions before lunch; in the first, on pharmaceutical applications, Dr R. L. Tranter (Glaxo Operations [UK] Ltd) addressed the possibilities of automated sample processing in the pharmaceutical industry, with particular reference to those analyses where the analyte is unstable and pre-preparation of batches for subsequent automated analysis is impossible. Dr A. W. Mace (Ciba-Geigy Ltd) then considered the use of a variety of preparative devices, such as the Technicon Solid-Prep sampler, for drug extraction, and then described the sample-preparation units designed as part of a new flow-injection analysis system developed jointly by Ciba-Geigy and Hamilton called AMICA (Automated Modules for Industrial Control Analysis). Finally, Mr D. Walker (ICI Pharmaceuticals Division) discussed problems of dissolution-rate studies and described the progress that has been made in automation of this essential quality-control procedure.

In the final session on inorganic applications, Mr G. Hawickhorst (Herzog Maschinenfabrik GmbH), in his keynote lecture, discussed the problems and possibilities of automation of sampling, sample preparation and presentation to X-ray fluorescence and optical emission spectrometers used as on-line process controllers in the cement and steel industries. Mr G. E. Purdue reviewed the general policy of automation of XRF spectrometry at the BP Research Centre, and, in the final paper, Dr J. Ottaway (Strathclyde University) described an automated electrothermal atomization/AA spectrometer system which he had developed to sample and analyse ultra-trace levels of toxic-metal airborne particulates; direct analysis of the particulate being achieved after its impaction onto the graphite tube of the ETA-AA analyser.

At the end of each session the speakers participated in interesting and often lively discussions on such items as the commercial availability of robots, the possibilities of incorporating optical and pressure sensors in robots 'hands', the respective merits of dynamic and passive sampling of organic vapours in the atmosphere and prospects for automated processing of the latter, accuracy and precision of automated as opposed to manual sample processing, and the relative merits of AA and inductively-coupled plasma emission spectroscopy for the analysis of airborne particulates.

In addition to the scientific programme a full social programme had been organized and was favourably received involving, as it did, a guided floodlit walk round the historic centre of York, a reception amongst the reconstructed streets exhibited in the Castle Museum, and a golf tournament over the championship course at Fulford followed by a tournament dinner.

The general feeling was that the symposium had been both a scientific and social success and had been much appreciated by the 70-odd participants.

**Clive J. Jackson**

*Occupational Hygiene Laboratory, 403 Edgware Road, London NW2 6LN*

### READER ENQUIRY SERVICE

Each advertisement and each editorial item in the Product News section in *Journal of Automatic Chemistry* carries a number which corresponds with a number on the Reader Enquiry Card bound into every issue.

If there are any items on which you would like further information, circle the relevant number on the card, indicate the volume and issue number, fill in your name and full address, and return the card to Taylor & Francis Ltd. Your card will then be forwarded to the company/ies concerned for action.

*Through using the Reader Enquiry Service, you can obtain information on a variety of products through one simple operation.*

### General Handbook of On-line Process Analysers

D. J. Huskins (Ellis Horwood Ltd, Chichester, UK, 1982), pp. 239, £30·00. ISBN 085312-329-2.

This book is the first of a series of five books being written by D. J. Huskins. Books two to five will be specifically devoted to descriptions of the various methods available for process control and their applications. Some applications are given in this volume, which also outlines the various abbreviations and symbols used throughout the series. It does not, however, contain any detailed descriptions of the analysers. Its intention is to give the reader a better understanding of the proper and correct use of analysers. In this aim it succeeds to a considerable degree.

As one has come to expect from Ellis Horwood Ltd, this book is extremely well produced and presented in an attractive cover. That its cost of £30 is relatively high is more a reflection on the economics of scientific publications on this scale rather than a true value of this book. 239 pages for £30 is not a very attractive proposition to the scientist wishing to purchase his own copy of this text, but it is at this level rather than as a library reference-book that this volume has its real value.

The volume provides an ideal introduction to the scientist embarking on a career in process instrumentation. It has a large section devoted to estimation of errors in calibration and standardization. Also stressed in some detail is the need for a precise specification of the process requirement. The similarity here between process instrumentation and automatic analysers for laboratory or clinical use is noticeable, since both need a reliable specification in detail of the analysis required and the objective of providing the data. Perhaps the only improvement that I would like to see in this volume is a more detailed and better explained section on cost justifications. In the small section devoted to costing—there are only four pages—some of the costings seem extremely low. It is becoming more important in today's economic climate to provide a true justification of large capital expenditure.

On the whole, the book is a valuable addition to the scientific literature and scientists are encouraged to purchase it.

**P.B.S.**

(This review was first published in *Talanta*.)

### EDITORIAL NOTE

The Editor is pleased to receive new books on chemical automation and mechanization. Review copies should be sent to:

*Dr Peter Stockwell, Plasma-Therm Ltd, Unit 3, 2/3 Kangley Bridge Road, Lower Sydenham, London SE26 5AR.*

